# Unraveling the Clinicopathological Diversity and Subtypes of Rhabdomyosarcoma: A Study From a Tertiary Care Center

**DOI:** 10.7759/cureus.54341

**Published:** 2024-02-17

**Authors:** Harika Mandava, Inuganti Venkata Renuka, Sudhakar Ramamoorthy

**Affiliations:** 1 Pathology, NRI Medical College, Chinakakani, IND

**Keywords:** myo d1, spindle cell rhabdomyosarcoma, pleomorphic rhabdomyosarcoma, alveolar rhabdomyosarcoma, embryonal rhabdomyosarcoma, rhabdomyosarcoma (rms)

## Abstract

Background and objective

Rhabdomyosarcoma (RMS) is a rare and malignant mesenchymal tumor characterized by skeletal muscle differentiation. While it is a common soft tissue sarcoma in children, its incidence significantly decreases with advancing age, rendering it exceptionally rare in individuals aged more than 45 years. This study aimed to shed light on the clinicopathological diversity and subtypes of RMS, thereby providing a comprehensive overview for enabling diagnostic precision and therapeutic strategies in treating this infrequently encountered malignancy in adults.

Methodology

This was a hospital-based cross-sectional study conducted in the Department of Pathology. Patients who were diagnosed with RMS over a period of three years were included in the study. The demographic features such as age and sex and aspects related to the tumor site, size, subtypes of RMS, and immunohistochemical expression were studied.

Results

A total of 14 cases were included in our study. The age at diagnosis ranged from four months to 65 years with a male-to-female ratio of 1:2.5. The sites of presentation were head and neck, trunk, pelvis, genitourinary tract, and retroperitoneum. The histological types were embryonal, alveolar, pleomorphic, and mixed and spindle cell types. The tumor cells were positive for immunohistochemistry markers desmin, MyoD1, and vimentin.

Conclusion

This study delved into the clinicopathological intricacies of RMS, offering comprehensive insights into its diverse subtypes. Our findings underscore the unique presentation of RMS in adults, with trunk and genitourinary tracts emerging as primary sites and alveolar and pleomorphic RMS observed as the predominant histological subtypes. Furthermore, the study sheds light on rare subtypes with distinct anatomical distributions.

## Introduction

Rhabdomyosarcoma (RMS) is a rare malignant soft tissue tumor associated with skeletal muscle differentiation [1]. It is commonly seen in children but rarely encountered in adults. This tumor accounts for about 3-4% of all childhood cancers [2]. Males are affected more commonly than females with a ratio of approximately 1.5 to 1.0 [3]. RMS shows striking diversity in terms of location, clinical presentation, and histologic subtypes. They may arise anywhere in the body, and each histological subtype has a site predilection [2]. According to the recent WHO classification, the various histological subtypes of RMS include embryonal (ERMS), alveolar (ARMS), pleomorphic RMS (PRMS), and spindle cell/sclerosing subtype [4].

The primary objective of this study is to provide a comprehensive overview of demographic information and pathological findings related to RMS. This includes an in-depth examination of clinical features, diverse anatomic locations, histologic subtypes, and immunohistochemistry (IHC) data associated with RMS.

## Materials and methods

Study design and setting

This was a hospital-based cross-sectional study conducted in the Department of Pathology after obtaining approval from the Institutional Ethics Committee. The prime objective was to conduct an in-depth analysis of RMS cases diagnosed in-house over a three-year period, aiming to provide a comprehensive overview of demographic information and pathological findings associated with this soft tissue neoplasm. Patient confidentiality and privacy were strictly maintained throughout the study. All cases of RMS diagnosed in-house were included in the study, ensuring a comprehensive representation of the patients within the hospital setting. Cases lacking sufficient clinical information and those without immunohistochemical correlation were excluded. A total of 14 RMS cases were included in the final analysis after applying the exclusion criteria.

Data extraction and analysis

The data extraction involved a meticulous review of medical records, encompassing clinical presentations, imaging findings, gross observations, and histopathological details. Parameters such as patient age, gender, tumor site, tumor size, histological subtypes, and IHC data were systematically recorded. Hematoxylin & Eosin (H&E)-stained slides and IHC slides were rigorously reviewed by experienced pathologists. Histological subtypes were classified according to the recent WHO classification. Descriptive statistics were employed to summarize the demographic and pathological characteristics of the RMS cases. The median age, gender distribution, anatomical locations, tumor sizes, and prevalence of different histological subtypes were analyzed to provide a comprehensive overview of the study cohort.

## Results

The age of the patients in this study ranged from four months to 65 years, with a median age of 21 years. The male-to-female ratio was 1:2.5. Tumors manifested in diverse anatomical locations including the head and neck, trunk, pelvis, genitourinary tract, and peritoneum. Some tumors exhibited uncommon histological subtypes and atypical anatomical locations, as detailed in Table 1.

**Table 1:** Distribution of cases based on age, sex, location, and histological type RMS: rhabdomyosarcoma

Serial no.	Age	Sex	Location	Histological subtype
1	65 years	F	Retroperitoneum	Alveolar RMS
2	60 years	F	Trunk	Alveolar RMS
3	1 year	M	Genitourinary (para testis)	Spindle cell RMS
4	49 year	F	Genitourinary (uterus)	Alveolar RMS
5	4 months	M	Pelvis (sacrococcygeal mass)	Mixed embryonal and alveolar RMS
6	19 years	F	Head and neck	Embryonal RMS
7	61 years	F	Trunk	Pleomorphic RMS
8	30 year	M	Retroperitoneum	Pleomorphic RMS
9	24 years	F	Head and neck	Embryonal RMS
10	15 years	M	Scrotal swelling	Embryonal RMS
11	32 years	F	Trunk	Pleomorphic RMS
12	20 years	F	Head and neck	Alveolar RMS
13	13 years	F	Genitourinary tract	Mixed embryonal and alveolar RMS
14	22 years	F	Trunk	Pleomorphic RMS

**Figure 1:**
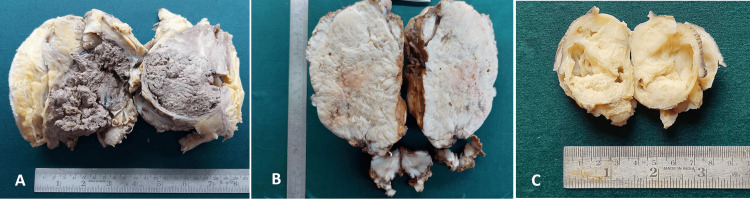
Dimensions of the tumors A. Gross photography of the subcutaneous mass in the left flank measuring 17 x 13 x 8 cm. The cut surface revealed a circumscribed unencapsulated, friable, grayish-brown tumor with hemorrhage and necrosis. B. Gross photography of the parotid mass measuring 18 x 15 x 10 cm with firm, yellow, heterogenous cut surface and patchy necrosis. C. Gross photography of a subcutaneous sacrococcygeal mass measuring 6.5 x 3.5 x 3 cm. The surface exhibits a yellowish-white solid and cystic areas

The dimensions of the tumors at their highest varied from 2 cm to 18 cm, as showcased in Figure 1.

**Figure 2:**
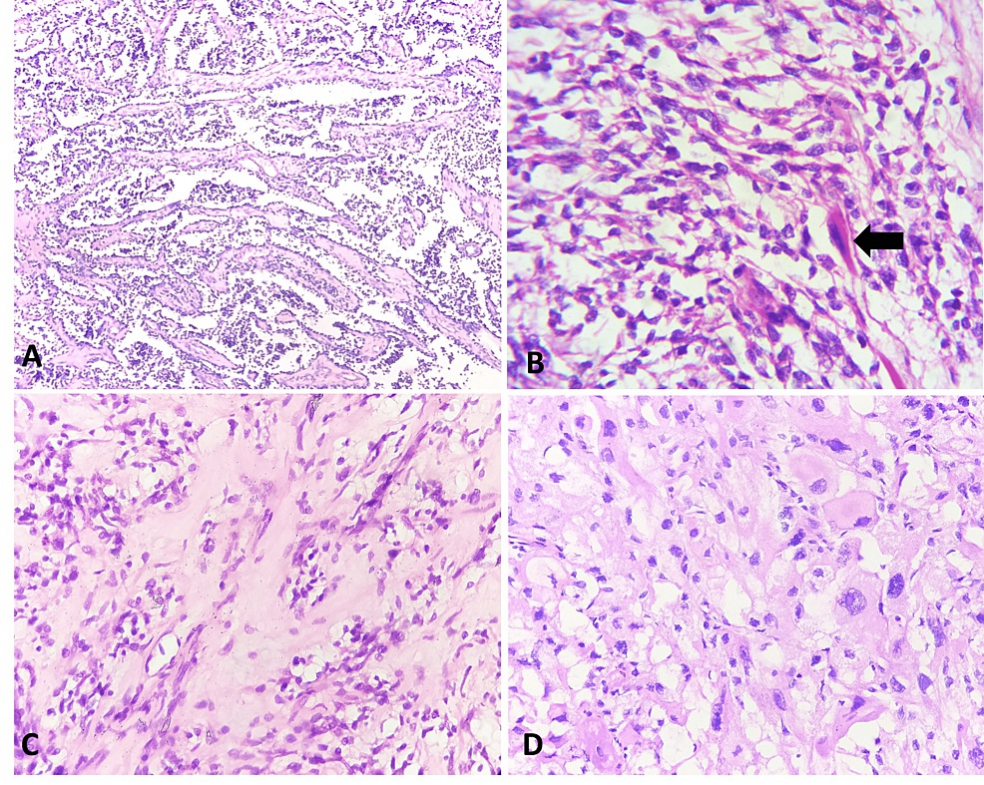
Histological analysis A. Low-power magnification of alveolar RMS exhibits a prominent alveolar pattern lined by tumor cells with round nuclear morphology, mild anisonucleosis, and scant cytoplasm (H&E, 100x). B. High-power magnification of embryonal RMS exhibits tumor cells with round to spindle-shaped hyperchromatic nuclei with scant cytoplasm and an occasional rhabdomyoblast (black arrow) (H&E, 400x). C. High-power magnification of spindle cell/sclerosing RMS shows spindle-shaped tumor cells with elongated nuclei, eosinophilic cytoplasm, and intervening sclerotic stroma (H&E, 400x). D. High-power magnification of pleomorphic RMS reveals pleomorphic tumor cells with pleomorphic nuclei, few with prominent nucleoli and abundant eosinophilic cytoplasm (H&E, 400x)

Histologically, the tumors presented as embryonal, alveolar, pleomorphic, spindled, and mixed types, as illustrated in Figure 2. Alveolar RMS and pleomorphic RMS emerged as the most prevalent histological subtypes.

**Figure 3:**
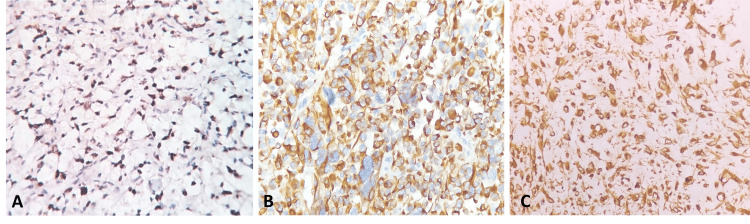
Immunohistochemistry A. Myo D1 immunohistochemistry shows diffuse nuclear expression in tumor cells. B. Desmin immunohistochemistry shows strong diffuse cytoplasmic positivity in tumor cells. C. A case of embryonal RMS, exhibiting diffuse strong cytoplasmic expression of vimentin in tumor cells

Immunohistochemistry revealed positivity for desmin, MyoD1, and vimentin, while negative for smooth muscle actin (SMA), as depicted in Figure 3. 

## Discussion

RMS is a malignant neoplasms that show morphologic, immunohistochemical, ultrastructural, or molecular genetic evidence of primary skeletal muscle differentiation [5]. Typically, these tumors manifest most prevalently as soft tissue sarcomas in childhood and adolescence, with a usual onset before the age of 20 years [6,7]. However, their occurrence is uncommon in adults and exceptionally rare beyond the age of 45 years [2,8]. Our study, in contrast with the general consensus, predominantly comprised an adult population with a mean age of 38.2 years, i.e., 10/14 cases (71.4%). Notably, a pronounced gender disparity was observed in our findings, with females outnumbering males at a ratio of 2.5:1. This contrasts with the studies by Rekhi B et al. [9] and Ahmad Z et al. [10] where males constituted the predominant population. These differences underscore the importance of considering age and gender variation in the epidemiology of RMS.

The gross size of the tumors ranged from 2 to 18 cm, with the most common sites being the trunk and genitourinary tract in the current study. This contrasts with other international studies by Turner and Richmon [11] and Ahmad et al. [10] where the most common site was the head and neck. This variation can be due to the underrepresentation of the pediatric population in our study as head and neck RMS is more common in the pediatric age group in other studies. The most common histological subtypes in our study were alveolar and pleomorphic RMS, which comprised 28.5% (4/14 cases) each. This was followed by embryonal at 21.4% (three cases). These findings align with those reported by Rekhi et al. [9] but differ from the results observed by Ahmad Z et al. [10]. This variation could be attributed to the disproportionately high numbers of elderly individuals in our study as pleomorphic RMS is the predominant histologic subtype in the elderly.

Among the histologic subtypes, the distribution of embryonal RMS in this study was predominantly seen in the head and neck region [parameningeal (one case) and parotid gland (one case)] and in the first and second decades of life. This aligns with the findings of Rekhi et al. [9] and Radzikowska, et al. [12]. Another case of embryonal RMS presented as a scrotal swelling in a 15-year-old child. Histologically, embryonal RMS was composed of round to spindle-shaped undifferentiated cells with hyperchromatic nuclei and myxoid stroma. In our study, alveolar RMS cases were seen in patients after the second decade of life, similar to the studies by Rekhi et al. and Ahmed et al. [9,10]. The anatomic distribution of these cases varied. There was a case of primary uterine RMS in our study, in a 49-year-old female, which is rare. Choi et al. also reported a similar case in a 90-year-old [13]. Histologically, alveolar RMS was composed of round to oval cells arranged in an alveolar pattern lined by fibrous septa with wreath-like giant cells.

In the present study, pleomorphic RMS was seen after the second decade of life and it is the most common variant in adults. This is comparable to studies by Stock et al. [14] and Ahmad et al. [10]. Among the four pleomorphic RMS cases in our study, three were in the trunk, and one in the retroperitoneum. The tumor was composed of haphazardly arranged pleomorphic to polygonal tumor cells with pleomorphic nuclei, some with prominent nucleoli and abundant eosinophilic cytoplasm. There were two mixed histological types, i.e., mixed alveolar and embryonal variants, one presenting as a sacrococcygeal mass and the other as a pubic mass. Histologically, these tumors showed a mixed pattern comprising tumor cells in alveolar pattern and sheets of round to oval cells. We also had a case of sclerosing spindle cell RMS of the para-testis. A study by Sarah et al. [15] showed that the most common site for spindle cell RMS is the para-testis. Histologically, the tumor cells were arranged in fascicles and whorls composed of spindle-shaped uniform cells with elongated spindle nuclei with inconspicuous nucleoli and eosinophilic cytoplasm with areas of hyalinization.

All RMS cases in our study exhibited strong positive staining for desmin and MyoD1, while embryonal RMS specifically displayed positivity for vimentin. Notably, these tumors were consistently negative for SMA. The IHC profile observed in our study aligns with the findings reported in a study by Ahmad et al. [10], indicating a consistent pattern of immunoreactivity across different cohorts.

## Conclusions

Our study engaged in a comprehensive exploration of various subtypes of RMS. The most common sites of occurrence in our study were the trunk and genitourinary tracts, with alveolar RMS and pleomorphic RMS emerging as the most prevalent histological subtypes. Notably, we have also identified rare histologic subtypes exhibiting diverse anatomical site distributions. However, it is crucial to interpret our findings in the context of certain limitations. The observed variations in age distribution and histologic subtypes, deviating from the general consensus, can be attributed to the relatively small sample size and the underrepresentation of the pediatric population in our study.
